# Seasonal and cultural effects on calendar day variations in trauma incidence in Japan

**DOI:** 10.1038/s41598-025-27973-z

**Published:** 2025-12-18

**Authors:** Keisuke Suzuki, Akira Endo, Tomohiro Akutsu, Hiromasa Hoshi, Akira Suekane, Ryo Yamamoto, Kazuma Yamakawa, Shin Watanabe, Akihiro Hirakawa, Yasuhiro Otomo, Koji Morishita

**Affiliations:** 1 Department of Acute Critical Care Medicine, Tsuchiura Kyodo General Hospital, 4-1-1 Otsuno, Tsuchiura, 300-0028 Ibaraki Japan; 2 Trauma and Acute Critical Care Medical Center, Institute of Science Tokyo, 1-5-45 Yushima, Bunkyo-ku, Tokyo, 113-8510 Japan; 3 Department of Emergency Medicine, Miyazaki Prefectural Miyazaki Hospital, 5-30 Kita-Takamatsu-cho, Miyazaki-shi, Miyazaki, 880-8510 Japan; 4 Department of Emergency and Critical Care Medicine, Keio University School of Medicine, 35 Shinanomachi, Shinjuku-ku, Tokyo, 160-8582 Japan; 5 Department of Emergency and Critical Care Medicine, Osaka Medical and Pharmaceutical University, 2-7 Daigaku-machi, Takatsuki-shi, 569- 8686 Osaka Japan; 6 Department of Clinical Biostatistics, Graduate School of Medical and Dental Sciences, Institute of Science Tokyo, 1-5-45 Yushima, Bunkyo-ku, Tokyo, 113- 8510 Japan; 7 Department of Critical Care Medicine and Trauma, National Hospital Organization Disaster Medical Center, 3256 Midoricho, Tachikawa, 190-0014 Tokyo Japan

**Keywords:** Diseases, Medical research, Risk factors

## Abstract

Trauma incidence patterns are influenced by societal and cultural factors. However, research has mainly focused on short-term periods. This study examined daily trauma trends throughout the year to identify fluctuations aligned with lifestyle patterns under a relatively homogeneous ethnocultural context, using long-term nationwide trauma data. Data from patients with trauma recorded in the Japan Trauma Data Bank (2004–2021; *n* = 383,473) were retrospectively analyzed. Participants were grouped by transport date into 365 daily cohorts. Daily patient volumes, injury severity, suicide attempts, and mortality were assessed. Outliers were identified using negative binomial regression with periodic functions, logistic regression adjusted for trauma severity, and the Generalized Extreme Studentized Deviate test. Human behavioral patterns appeared to significantly influence the trauma incidence. Cases increased during Golden Week (late April–early May), Sports Day (October 10), Culture Day (November 3), and the end of the year while declining during the Obon holiday (mid-August) and in early January. Suicide attempts peaked in May–June and September, diverging from overall trauma trends. Mortality rates remained consistent, with no significant seasonal variation or outliers. Long-term data suggest that trauma volumes vary in relation to seasonal and cultural events, offering valuable insights for optimizing trauma resource allocation and preventive strategies.

**Supplementary Information:**

The online version contains supplementary material available at 10.1038/s41598-025-27973-z.

## Introduction

Trauma remains a leading cause of death and disability worldwide, placing a substantial burden on healthcare systems and society. Numerous epidemiological studies have investigated temporal fluctuations in the trauma incidence throughout the calendar year; however, most have focused on specific seasons^1^ or holidays within limited observation periods^2–16^. The impact of holidays—referred to as the “holiday effect”—on the trauma incidence is well established and remains a common research topic. Nevertheless, the broader influence of year-round lifestyle patterns on trauma occurrence has not been thoroughly explored due to the short-term scopes of previous studies.

Japan offers a favorable setting for such an investigation due to its relatively homogeneous population and shared cultural practices, which contribute to largely uniform behavioral patterns. For instance, Japan observes distinct periods in which daily routines deviate markedly from the norm, such as Golden Week (April 29–May 5), Obon (a mid-August holiday during which the spirits of deceased relatives are traditionally believed to return), and the year-end and New Year holidays (December 28–January 3). Additionally, seasonal variations in suicide have been documented, with numerous studies reporting a higher incidence during warmer months such as in spring and summer^17–22^. However, the impact of these temporal patterns has yet to be assessed using long-term, comprehensive data. Employing a day-by-day analytical approach to nationwide trauma data collected over 18 years, this study aimed to identify trends in the trauma incidence that extend beyond incidental annual fluctuations. These findings may contribute to the optimization of trauma care systems through more effective resource allocation and preventive strategies^23–26^.

## Methods

### Study setting

This retrospective observational study analyzed data from the Japan Trauma Data Bank (JTDB) covering the period from January 2004 to December 2021. Established in 2003, the JTDB is maintained by the Japanese Association for the Surgery of Trauma and the Japanese Association for Acute Medicine to monitor and improve the quality of trauma care in Japan. Participating institutions are required to register all patients with trauma who present with an Abbreviated Injury Scale (AIS) score ≥ 3 in at least one body region. As of March 2022, 303 hospitals participated in the JTDB, with approximately 75% classified as tertiary-level emergency centers, comparable to level 1 trauma centers in other countries^27^.

The JTDB captures 92 data elements pertaining to patients and hospitals, including demographics, the date and cause of injury, prehospital information, the mechanism of injury, suicide attempts, vital signs, AIS scores, the Injury Severity Score (ISS), the probability of survival based on the Trauma and Injury Severity Score, discharge status, and treatments administered in emergency departments and operating rooms. Data are entered into an online portal by the treating physicians or designated registrars at each hospital.

This study was conducted in accordance with the Declaration of Helsinki^28^ and its subsequent amendments. The study protocol received approval from the Ethics Committee of Tsuchiura Kyodo General Hospital. Given the retrospective nature of the study, the requirement for obtaining individual informed consent was waived. An opt-out approach was provided to allow patients the opportunity to decline participation.

### Data collection

The following variables were extracted from the JTDB: age, sex, date and cause of injury, classification of injury, ISS, AIS, and discharge outcomes.

### Study participants

All patients with trauma whose records included a complete injury date (year, month, and day) were included. Data from February 29 were excluded. Patients with missing ISS or unknown discharge status were excluded. Analyses were performed on a complete-case basis for these variables.

### Statistical analysis

Participants were categorized into 365 daily groups based on the date of hospital arrival. The number of daily admissions for each calendar day was determined by aggregating the raw observed counts over the entire study period. Daily mortality rates were also calculated. Both metrics were plotted over the calendar year (January 1–December 31). Patients with severe trauma, defined as ISS ≥ 16, were analyzed separately. Additional analyses were performed for patients with suicide attempts. To model seasonal fluctuations, negative binomial regression incorporating periodic functions (sine and cosine transformations of calendar days) was used due to overdispersion in count data. Standardized residuals (SRs) from the fitted model were computed for each calendar day; absolute SRs > 3.0 were considered statistically significant outliers. For mortality analysis, logistic regression was performed using a generalized linear model, incorporating the calendar date and ISS as independent variables. We applied the Generalized Extreme Studentized Deviate test at α = 0.05 to the ISS-adjusted daily mortality risk estimates—normality was assessed using the Shapiro–Wilk test. All statistical analyses were conducted using R version 4.2.2 (R Foundation for Statistical Computing, Vienna, Austria).

## Results

A total of 418,769 patients with trauma and complete injury dates were identified, of whom 35,296 (8.4%) were excluded due to missing ISS data or unknown survival status at discharge. Consequently, 383,473 patients were included in the primary analysis (Table 1, Supplementary Fig. S1).

**Table 1:** Demographic and clinical characteristics of patients with Trauma, stratified by injury severity and suicide Attempt.

	All patients	Patients with severe trauma	Patients who attempted suicide	Patients with severe trauma who attempted suicide
n	383,473	155,289	21,637	11,943
Female, sex, n, (%)	145,223 (37.9)	46,976 (30.3)	10,260 (47.5)	5,671 (47.6)
Age, Median (IQR)	64 [40, 79]	63 [41, 76]	42 [28, 9]	42 [28, 58]
Type of Trauma n, (%)				
Blunt	355,466 (92.7)	146,147 (94.1)	14,597 (67.5)	9,908 (83.0)
Penetrate	12,714 (3.3)	2,696 (1.7)	5,743 (26.5)	1,184 (9.9)
Injury Severity Score, Median (IQR)	10 [9, 20]	22 [17, 29]	17 [9, 29]	29 [22, 41]
Revised Trauma Score, Median (IQR)	7.84 [7.55, 7.84]	7.84 [5.97, 7.84]	7.11 [4.09, 7.84]	5.97 [0.00, 7.84]
TRISS score, Median (IQR)	0.97 [0.92, 0.99]	0.91 [0.69, 0.94]	0.96 [0.38, 0.99]	0.68 [0.04, 0.95]
Died, n (%)	36,959 (9.6)	32,418 (20.9)	6,001 (27.7)	5,489 (46.0)

Data are presented as medians (interquartile range) or numbers (%).

IQR, interquartile range.

TRISS score is presented as the calculated probability of survival using the TRISS methodology.

**Fig. 1:**
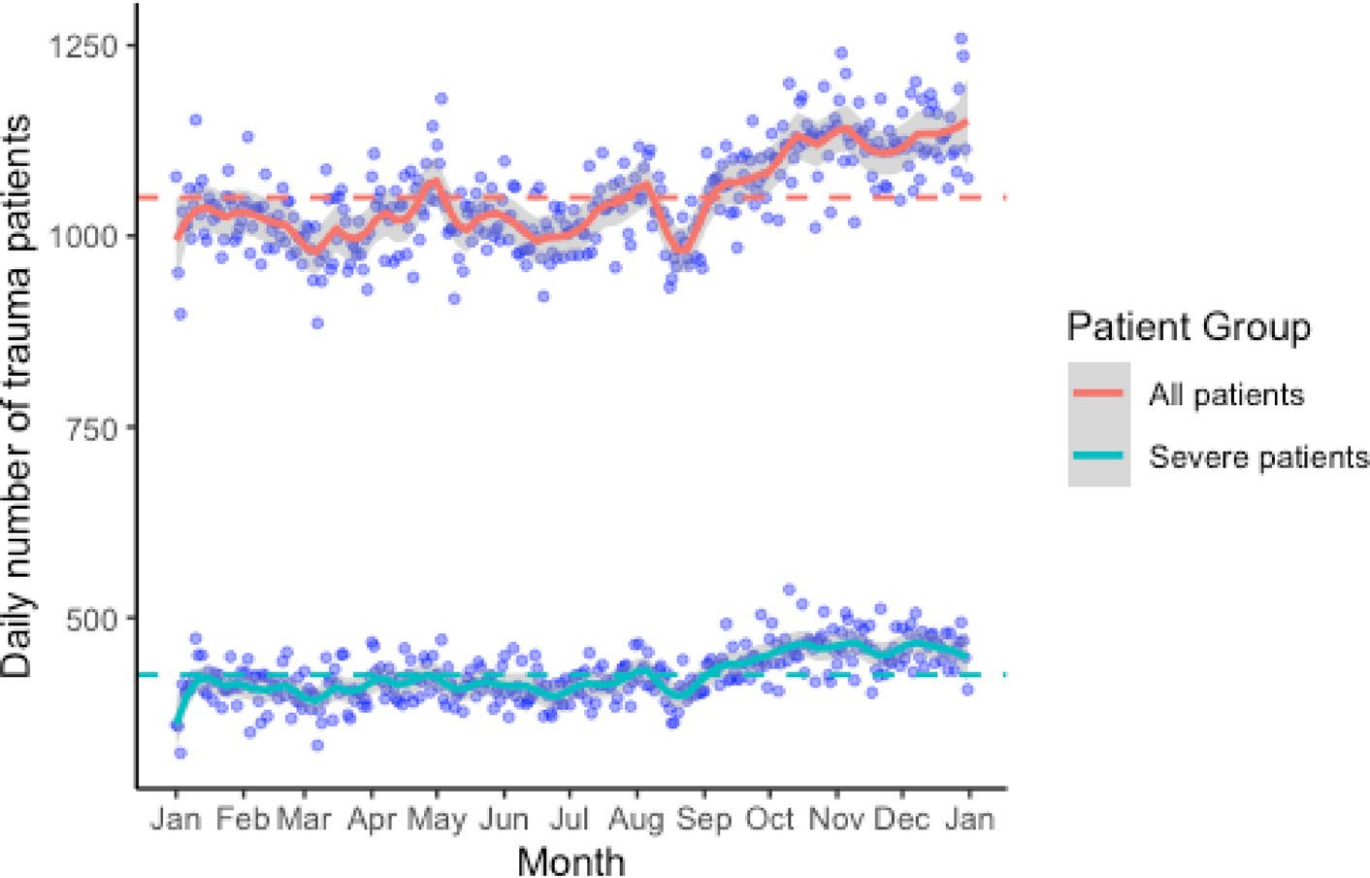
Trends in daily number of trauma cases and severe trauma cases. This figure displays the daily number of trauma cases in Japan from January 1 to December 31, based on data from 383,473 patients. Each point represents the number of patients on a given day. The red line indicates the overall trend, while the blue line represents patients with severe trauma (Injury Severity Score [ISS] ≥ 16). Notable peaks are seen during early May (Golden Week), during early August (summer vacation), around October 10 (Sports Day), in early November (Culture Day), and towards the year-end holidays, with the highest count on December 28 (1,259 patients). Significant decreases occurred during mid-August (Obon holiday), with the lowest count on March 7 (886 patients).

**Fig. 2:**
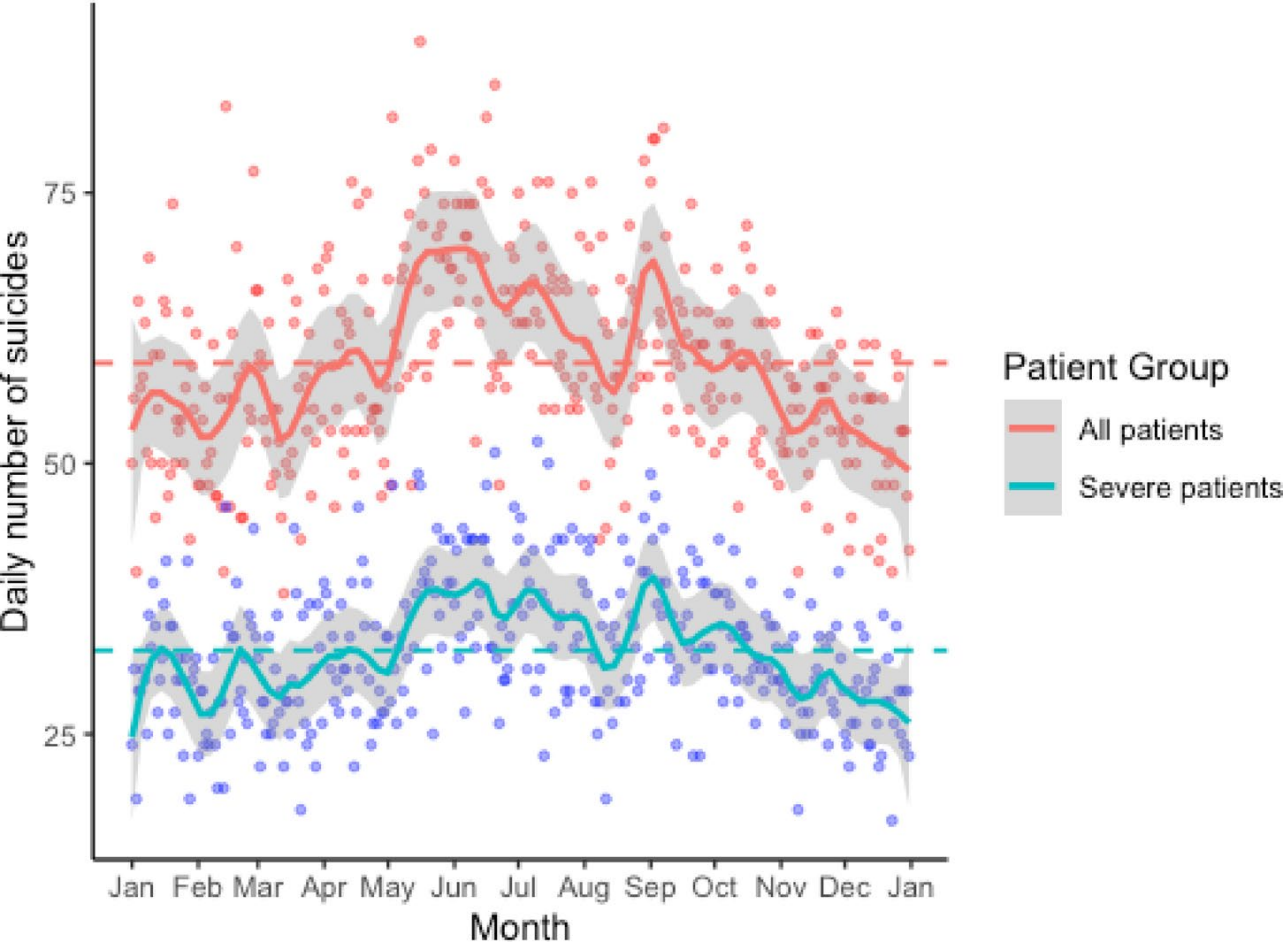
Trends in daily incidence of suicide attempts. This figure shows the daily incidence of suicide attempts in Japan from January 1 to December 31. Individual dots represent the daily number of cases of suicide attempts, with the red line showing the overall trend and the blue line showing cases of severe trauma resulting from attempted suicide (ISS ≥ 16). Both groups displayed similar seasonal patterns, peaking in May and decreasing in mid-August (summer holidays) and around the year-end holidays and New Year’s day.

Daily trauma case counts varied markedly throughout the year, with higher volumes generally observed from September to December. A statistically significant increase was also seen during Golden Week (April 29–May 5). Peak volumes occurred around the year-end (December 28–29) and on national holidays such as Culture Day (November 3) and Sports Day (October 10). In contrast, case volumes declined notably during the Obon period (mid-August), particularly around August 15, and reached their minimum on March 7 (886 patients), with the second-lowest count on January 3 (898 patients; Fig. 1). Negative binomial regression with periodic functions identified significant outliers on January 3 (SR: −3.58), May 3 (SR: 3.44), and December 28 (SR: 3.16), reflecting deviations from expected seasonal patterns (SR > 3.0). Additional dates with moderate deviations (SR > 2.0) are listed in Supplementary Table S1. In total, 155,289 patients experienced severe trauma (ISS ≥ 16). The annual trends for severe trauma cases mirrored the overall pattern, with the lowest count also occurring on January 3 (323 patients). Reductions were again prominent during the Obon and New Year holidays (Fig. 1). A distinct pattern was observed among the 21,637 patients (5.6%) who attempted suicide. Patient volumes increased during the period of May to June and from late August to September but showed a decreasing trend from October to December. Volumes also decreased during the Obon period and the year-end/New Year holidays. A similar seasonal trend was observed among patients with severe trauma due to suicide attempts (Fig. 2).

**Fig. 3:**
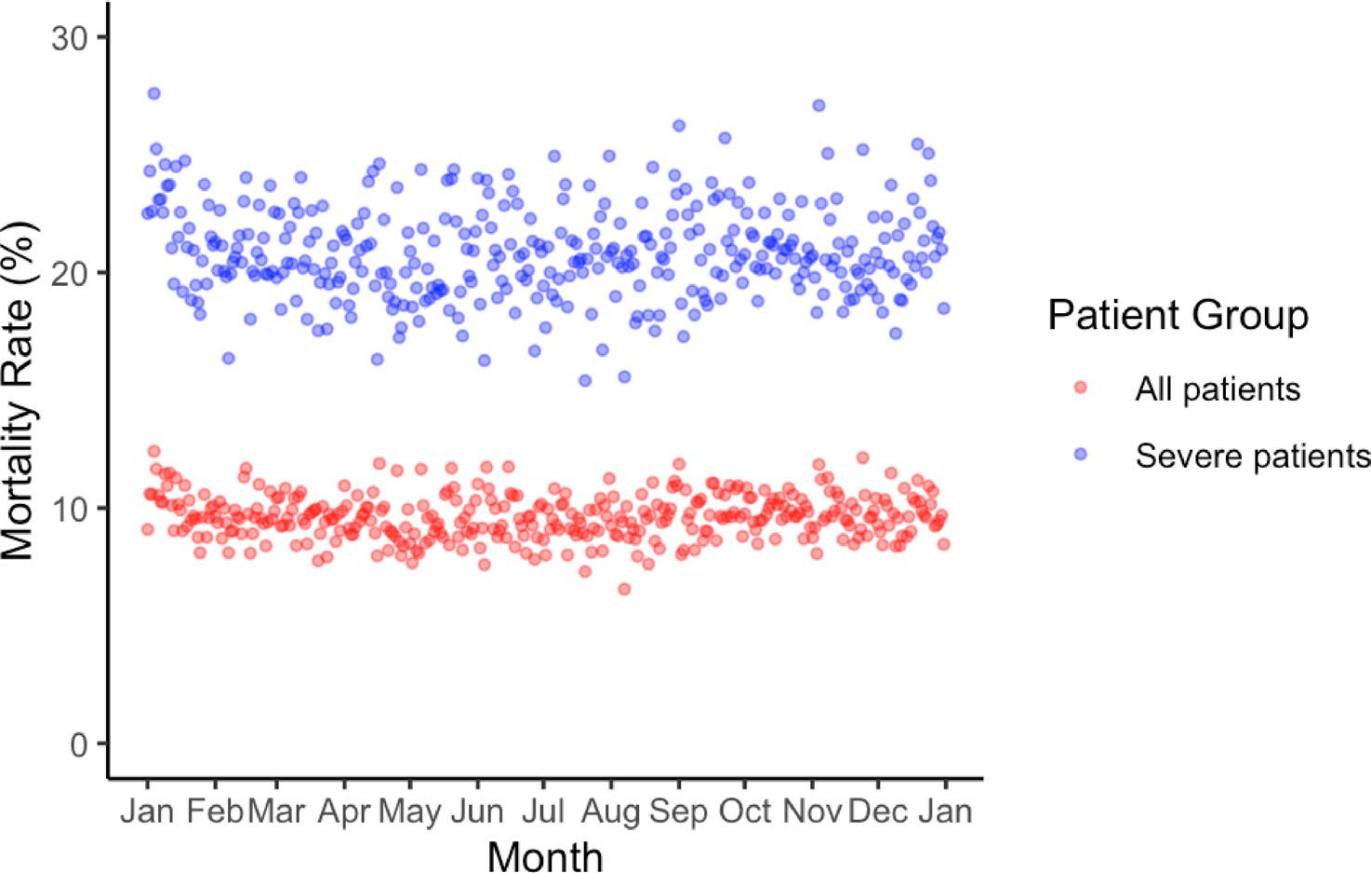
Daily mortality rates. Daily mortality rates are shown for each day of the year, divided into 365 days from January to December. The graph plots the daily mortality rates as blue dots, showing mortality rates with no significant outliers. The data were uniform over the entire period, with no days identified as outliers.

**Fig. 4:**
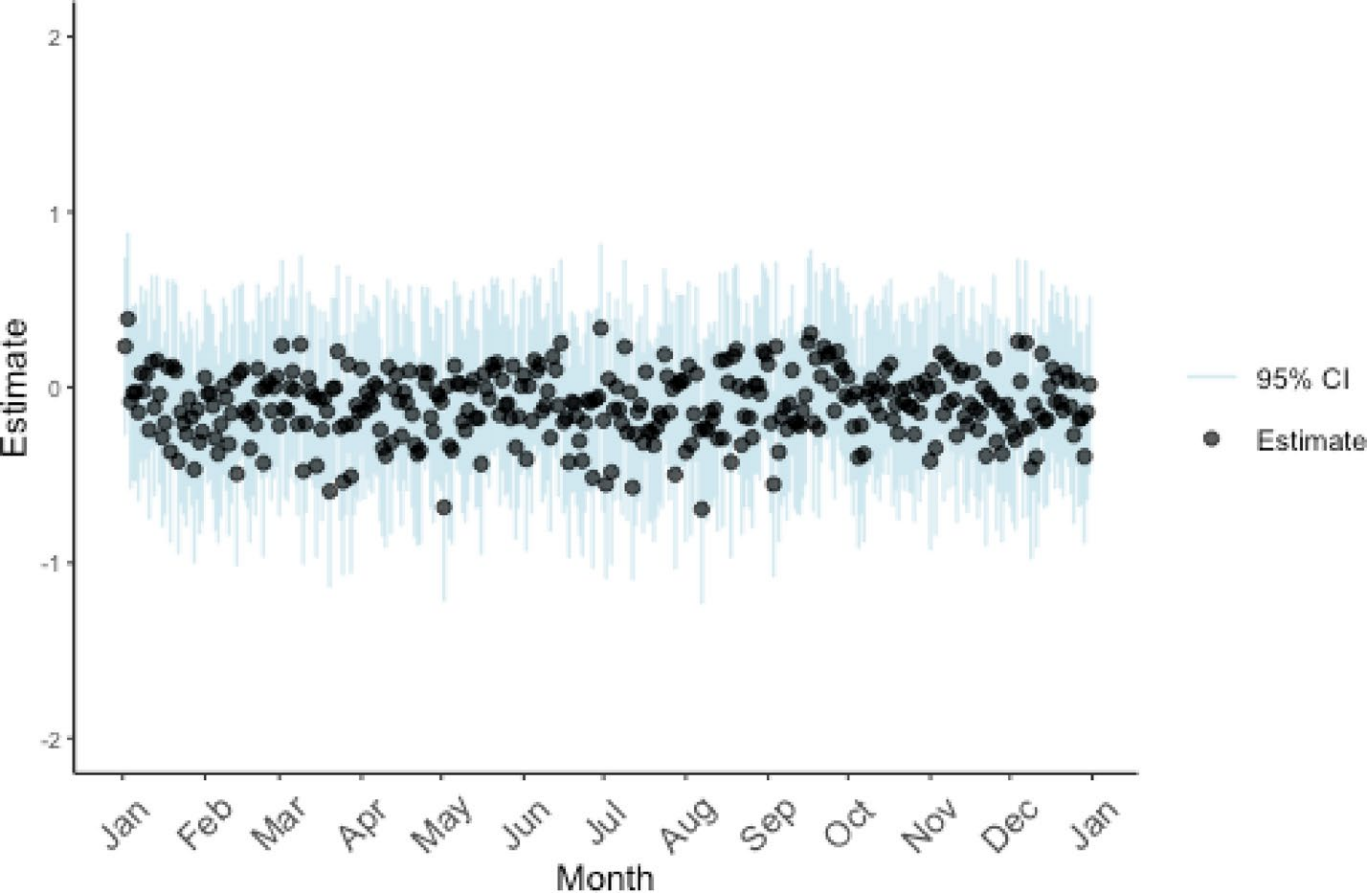
Trends in mortality risk in all patients, adjusted by Injury Severity Scores (ISS). Each point represents the daily mortality risk adjusted for trauma severity based on ISS scores. Each point reflects the adjusted mortality risk for that day. No statistical outliers were identified using the Generalized Extreme Studentized Deviate (GESD) test, suggesting a consistent mortality risk across the calendar year.

The average daily mortality rate was 9.6%, peaking on January 4 (12.4%) and reaching its lowest rate on August 7 (6.6%) (Fig. 3). Overall, daily mortality rates exhibited minimal fluctuations, without discernible seasonal trends or clustering of elevated or reduced mortality (Fig. 3). Applying the Generalized Extreme Studentized Deviate test to ISS-adjusted daily mortality risk estimates identified no statistically significant outlier days across the calendar year (Fig. 4). Annual trends in total trauma case counts exhibited generally consistent seasonal fluctuations throughout the entire study period from 2004 to 2021 (Supplementary Fig. 2).

## Discussion

This analysis of data from 383,473 patients with trauma over an 18-year period revealed distinct seasonal and cultural patterns in trauma volumes in Japan. Trauma incidence increased from late summer through autumn, particularly between September and December, while notable declines occurred immediately following New Year’s Day. Specific periods demonstrated characteristic spikes in trauma cases—particularly during Golden Week (April 29–May 5), Sports Day (October 10), Culture Day (November 3), and the year-end period. These increases are likely related to increased physical activity, travel, and recreational participation associated with national holidays. Conversely, trauma volumes significantly decreased during the Obon holiday in mid-August and the New Year period. Although less pronounced, the volume of patients with severe trauma followed a similar trend.

The principal strength of this study lies in its comprehensive, day-by-day analysis over an 18-year period. The relative homogeneity in lifestyle and cultural practices in Japan provides a unique opportunity to detect consistent temporal variations in the occurrence of trauma, which are often obscured in shorter-term or demographically diverse studies. While prior research has focused primarily on off-hour effects^14–16,29–33^ or broader monthly trends^34–36^, this study uncovered finer patterns linked to societal routines and cultural events.

Regarding holiday-related trauma patterns, in contrast to reports of increased trauma during holidays such as Christmas and New Year’s Eve, our data showed that trauma cases in Japan significantly decreased during its most culturally important vacation periods—the New Year holiday (which includes the lowest incidence day of the year on January 3) and the Obon holiday in mid-August. This decrease may reflect behavioral changes during these culturally significant times, such as a shift toward family gatherings. Furthermore, this study revealed that suicide-related trauma exhibited peaks (in May–June and September) that were distinct from those in the overall trauma fluctuation pattern. Although the seasonality of suicide attempts is widely known, its patterns are not uniform. While a general trend of a spring peak and a winter trough is observed in many regions, the specific shape and amplitude are said to vary depending on factors such as climate, demographics, and socioeconomic conditions^21^. In Japan specifically, a biphasic pattern with peaks in spring and autumn has been reported, attributed to psychosocial stress associated with turning points in social life, such as the start of the academic/fiscal year in April and the period after the summer vacation in September^22^. These peaks are considered to be a result reflecting this unique Japanese context.

A key finding of the present study is the consistency of mortality rates throughout the calendar year. In the adjusted mortality risk analysis, no statistical outliers were identified throughout the year. This stability suggests that trauma care delivery remained consistent across Japan regardless of patient volume fluctuations. Accordingly, the implementation of preventive strategies tailored to annual trauma fluctuations may be more effective in reducing trauma-related mortality than efforts solely aimed at redistributing medical resources. Our findings provide actionable data for the targeted implementation of preventive strategies. This data-driven approach may be more effective than solely redistributing medical resources. For example, the spikes in trauma during major holidays warrant targeted traffic safety campaigns before high-travel periods such as Golden Week and the year-end holidays. Similarly, the distinct peaks in suicide attempts suggest that enhanced community-based mental health outreach could be strategically timed to periods with elevated risk. Notably, in Japan, the Basic Act on Suicide Countermeasures stipulates a national Suicide Prevention Week in September and a Strengthening Month in March. Aligning these nationwide initiatives with the peaks observed in May–June and late August to September, as demonstrated in our study, may improve the appropriateness and timeliness of preventive efforts. Furthermore, these predictable patterns can inform hospital and emergency department readiness to better prepare for high-incidence periods.

This study also had several limitations. The analysis was limited to patients with AIS scores ≥ 3, which may have excluded less severe cases and introduced selection bias. While the JTDB includes approximately 75% of tertiary emergency hospitals, trauma trends at nonparticipating facilities remain unknown. Furthermore, the lower volume of cases in the early years of the registry (2004–2006) may reflect an initial period of under-registration. The results may not be generalizable to other countries due to Japan’s unique cultural calendar and societal practices. Although holiday dates shift slightly year to year, the long observation period likely mitigated the effects of this variation. External influences such as climate conditions, natural disasters, and social disruptions (e.g., the Coronavirus Disease 2019 pandemic starting in early 2020) may have also affected trauma patterns. Due to the study’s retrospective observational design, causal relationships between calendar events and trauma occurrence cannot be definitively established. Complete-case analysis excluded patients with missing ISS or survival data; however, the missingness appeared to be randomly distributed across the study period, likely minimizing its impact. Our outcome analysis was limited to mortality; other clinically important non-mortality outcomes, such as complication rates, length of stay, and discharge disposition, were not sufficiently included in this study and should be prioritized in future research. We also did not stratify trauma trends by the mechanism of injury, which could provide more detailed insights for tailoring prevention strategies. Moreover, center-level heterogeneity was not explicitly modeled, which may also have influenced the observed patterns.

In conclusion, trauma volumes in Japan followed consistent annual trends aligned with cultural holidays and seasonal routines. Suicide attempts exhibited a distinct pattern and did not substantially affect the overall trauma volume. No significant differences or outlier days were observed in mortality rates. These findings offer valuable insights for planning of trauma care resources and highlight the importance of behavioral and societal factors in trauma prevention. Future studies should explore the psychosocial drivers underlying these temporal variations.

## Supplementary Information

Below is the link to the electronic supplementary material.

Supplementary Material 1

## Data Availability

The data used in this study are not publicly available. Access was granted only to institutions participating in the Japan Trauma Data Bank (JTDB), in accordance with regulations set by the Japanese Association for Trauma Surgery and the Japanese Association for Acute Medicine.
