# Supplementary material for: Seasonal and cultural effects on calendar day variations in trauma incidence in Japan
Source: Sci Rep. 2025 Dec 18;15:44106. doi: 10.1038/s41598-025-27973-z (PMC12715191; doi:10.1038/s41598-025-27973-z)
Supplement: Supplementary file 1 — Supplementary Material 1 [file 41598_2025_27973_MOESM1_ESM.docx]

**Supplementary Information**

| **Supplementary Table S1**. Dates Identified as Outliers Based on Negative Binomial Regression Analysis Using Periodic Functions (Standardized Residuals > \|2.0\|) | | | |
| --- | --- | --- | --- |
| Date | Observed Patients | Standard Error | Standardized Residual |
| 01-03* | 898 | 1,079 | -3.58 |
| 05-03† | 1,180 | 1,000 | 3.44 |
| 12-28* | 1,259 | 1,083 | 3.16 |
| 03-07 | 886 | 1,026 | -2.85 |
| 12-29* | 1,236 | 1,083 | 2.77 |
| 04-29† | 1,144 | 1,001 | 2.76 |
| 01-02* | 952 | 1,080 | -2.49 |
| 11-03¶ | 1,240 | 1,102 | 2.46 |
| 08-16§ | 933 | 1,057 | -2.45 |
| 05-01† | 1,119 | 1,000 | 2.30 |
| 08-17§ | 944 | 1,058 | -2.24 |
| 08-31 | 958 | 1,070 | -2.18 |
| 08-25 | 962 | 1,065 | -2.01 |
| Negative binomial regression analysis incorporating periodic functions was performed to identify significant deviations in daily trauma patient numbers from expected seasonal patterns.  Standardized residuals (SRs) were calculated for each calendar day.  Days with absolute SRs > 3.0 were defined as significant outliers, and days with absolute SRs between 2.0 and 3.0 were additionally listed as notable deviations.  * New Year holidays  † Golden week  § Obon period  ¶ Culture Day | | | |

**Supplementary Figure S1**: Flow diagram of patient selection.

A total of 427,561 trauma patients were initially identified from the Japan Trauma Data Bank (JTDB) between April 2004 and December 2021. Of these, 8,792 patients were excluded due to missing date information or data recorded on February 29. Subsequently, an additional 35,296 patients were excluded due to missing data (18,476 patients with missing Injury Severity Score [ISS] and 24,314 patients with missing survival status at discharge). Consequently, 383,473 patients were included in the primary analysis.

**Supplementary Figure S2.** Annual Trends in Total Patients with Trauma (2004–2021).

**
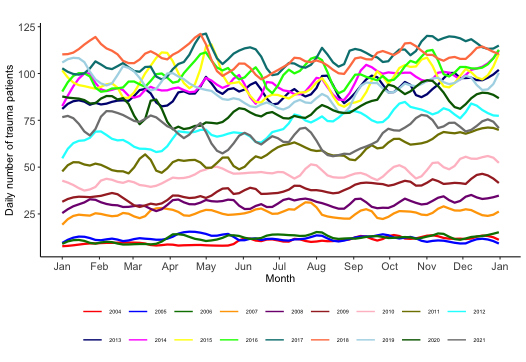
**

This graph depicts the annual trends in total patients with trauma over an 18-year period from January 1, 2004, to December 31, 2021. Each line signifies the pattern of patients for a single year, with color coding for distinction. Despite yearly variations, the data reveal consistent seasonal trends: a spike in patients during early May, a decline in mid-August, an increase from October through to the end of the year, and a decrease at the commencement of the new year. These trends provide insight into the recurrent seasonal effects on trauma patients in Japan. Analysis was conducted on a complete-case basis.
